# Synthesis and Properties of Polystyrene Composite Material with Hazelnut Shells

**DOI:** 10.3390/polym15153212

**Published:** 2023-07-28

**Authors:** Natalia Igorevna Cherkashina, Zoya Vladimirovna Pavlenko, Dar’ya Vasil’yevna Pushkarskaya, Lyubov Vasilievna Denisova, Semen Nikolayevich Domarev, Dar’ya Aleksandrovna Ryzhikh

**Affiliations:** Department of Theoretical and Applied Chemistry, Belgorod State Technological University Named after V.G. Shukhov, 308012 Belgorod, Russia; pavlencko.zoe@yandex.ru (Z.V.P.); dashamenzhulina@mail.ru (D.V.P.); loveden13@mail.ru (L.V.D.); domarev542@gmail.com (S.N.D.); sinebokd@mail.ru (D.A.R.)

**Keywords:** hazelnut shell, composite material, modification, polystyrene matrix, water absorption

## Abstract

In this study we evaluated the potential use of hazelnut shell powder in the production of a composite material. Polystyrene was used as a polymer matrix. This work presents the results of modifying hazelnut powder particles to create a polystyrene shell on their surfaces. Modification of the filler increased its contact angle wetted with water from θ=60.16±1.03° to θ=87.02±1.10°. Composite materials containing from 10 to 50 wt.% of modified hazelnut shell powder were prepared and studied. As a result of the experiments, it was found that the composites have optimal physical, mechanical, and operational properties at the following ratio: polystyrene 60–80 wt.%, modified hazelnut shell powder 20–40 wt.%. If the introduction of polystyrene was more than 90 wt.%, the flexural strength and Vickers hardness were quite low at the load of 200 g, and accordingly, the durability of such materials was not satisfactory. These samples are characterized by small percentages of hazelnut shells; therefore, the resulting material will be of pale, unsaturated color. The upper limit of the working temperature range for the composite lies between 265.0–376.0 °C, depending on the percentage of the hazelnut shell powder filling.

## 1. Introduction

Global trends in ecologization result in the search for new “green” materials, rapidly renewable resources with low cost, which are not toxic, and will not be inferior in properties to synthesized, artificial materials; they will contribute to maintaining the ecological balance in the world and solve the problem of their disposal [1,2]. “Green composites” are created by homogenizing substances based on polymeric matrices and fillers made of plant wastes (shells, husks, fibers, kernels); it is a step towards replacing synthetic fibers with natural ones in compositions [3,4]. Agro-industrial wastes attract special attention. They are disposed of in large quantities and are not reused in other fields [5], although this provides environmental and economic benefits [6]. In the process of a closed-loop economy, the possibility of recycling agricultural wastes is of particular importance [7,8]. They can give composite materials the necessary aesthetic appearance and adjust and set certain parameters, while contributing to the preservation of the forest belt and maintaining a high level of physical and mechanical characteristics of the obtained materials, since in some areas in the future the demand may exceed the supply of this resource, and its recovery takes dozens of years [9,10]. Therefore, composites based on plant wastes are becoming increasingly popular [11,12,13,14], as they replace expensive materials, while maintaining high quality characteristics, and are used in various fields [15,16]: in food packaging [17,18,19]; in the aerospace and automobile industries to reduce the weight of certain internal parts [20,21]; in construction industries to reduce cost and to create new, environmentally friendly materials; and to partially replace fine filler in concrete and other areas of production [22,23,24]. 

Hazelnut shells are available in large quantities during the harvest season. They are a by-product, an agricultural waste product that can be used as a renewable raw material for production. Hazelnut shells are a versatile, renewable resource that can be used for the synthesis of high-quality products and plant-based polymers, while having a high degree of hardness and impact resistance. Worldwide, in the harvesting season the hazelnut yield is about 530 thousand tons [25]. The shells are almost 70% of the total mass and are of no value in reuse. Since the hazelnut shells are of low value, scientists have not discovered the full potential of this waste so far. It is used as a heat source, as a solid biofuel, or as a raw material for the production of furfural [26,27,28], although the area of its application can be significantly expanded. In the study [29], hazelnut shells were used as a filler for a composite with the addition of polylactic acid. Their thermomechanical and physical properties were studied. The results showed that the addition of hazelnut shells had little effect on the results, but increased the crystallinity index, which positively affects the size stability of the composite. In other studies [30], this author added epoxidized linseed oil to the polymer matrix and hazelnuts to reduce the plasticity index, which generally improves the overall properties of the composite with polylactic acid and hazelnut shells. In their work [31], Aliotta L. and others studied a synthesized composite based on PLA3251D with the addition of hazelnut shells; the resulting material shows decreased viscosity in the molten state and a low level of tensile strength. Pradhan P. and others [32] studied the physical and mechanical properties of composites with a walnut shell filler. In spite of the high content of walnut shells, density and porosity parameters of the composites remained high enough. Kufel A. and others [33] studied physical and mechanical properties of hybrid composites based on polypropylene with the addition of hazelnut shells and basalt fiber, with maleic anhydride. The resulting material is characterized by improved physical and mechanical properties, but rather low moisture absorption. Ceraulo M. and others [24] researched the rheological properties of Bi^®^ El51N0 polyester composites with the addition of hazelnut shells, specifically their mechanical and morphological properties, to obtain environmentally friendly biocomposites. At moderate concentrations, the stiffness and impact toughness of the material improve, and the biocomposite is easily recyclable. 

Even though there exist many works studying this topic, in most of them a relatively low content of hazelnut shells is introduced due to its unsatisfactory compatibility with other constituents of the polymer matrix.

Polystyrene (PS) is widely used in the construction industry in insulating materials and noise-absorbing screens, in medicine, in utilities, and in the food industry. Polystyrene is known to be poorly biodegradable [34]. Therefore, it is promising to use it for composite materials, for which, on the contrary, resistance to non-decomposition in natural conditions is important. For example, based on polystyrene, it is promising to create composite materials that can be used to create building materials for outdoor and garden furniture. Benchouia et al. [35] have developed a new insulating material based on date palm fibers and polystyrene. The replacement of one third of the composition with the proposed composite showed the promise of such applications as thermal insulation, with a decrease in thermal conductivity of up to 50% [35]. Onifade et al. [36] showed the possibility of using reinforced biochar from psyllium stalk fibers to reinforce a polystyrene composite. The eco-friendliness of the composite gives a better solution to agro-waste disposal rather than burning [36]. Adeniyi et al. [37] developed a technology for the production of polystyrene composite with wood dust (isoberlinia doka). With the addition of wood dust in the amount of 30%, an improvement in the mechanical and thermal characteristics of the composite was observed [37].

Despite many studies on the creation of composites based on polystyrene and vegetable fillers, there are no works on the synthesis of a polystyrene/hazelnut shell composite.

The objectives of the study were to increase the compatibility of hazelnut shells with the polystyrene for greater incorporation of the shell into the matrix and to create highly filled composites. The use of rigid components of polystyrene and hazelnut shells will make it possible to create a composite material with high mechanical properties necessary for use as materials for garden furniture.

## 2. Materials and Methods

### 2.1. Materials 

In this study we used Polystyrene 525 (producer PJSC Nizhnekamskneftekhim, Nizhnekamsk, Russia) as a polymer matrix. Table 1 shows the main significant properties of the polymer used. 

Hazelnut shells were used as a filler. Shell powder was obtained by grinding raw materials (hazelnut shells from the harvest of 2022) in vibratory and planetary mills.

Toluene (LLC Component-Reaktiv, Moscow, Russia) was used as a solvent to modify hazelnut shells. Table 2 shows the main properties of toluene.

### 2.2. Hazelnut Shell Modification 

Hazelnut shells were preliminarily ground in WM3 vibrating mill (LLC CONSIT Holding, Moscow, Russia) for 3 min, then in XQM-1An planetary mill (Jiangxi Victor International Mining Equipment Co., Ltd., Shichen, China) for 60 min. Then there was rinsing with distilled water and drying in a BINDER oven (Binder, Tuttlingen, Germany) for 60 min at 150 °C. Afterwards, the ground shells were sieved through a 64 µm sieve.

The filler was modified by creating a polystyrene coating on its surface in order to impart a hydrophobic finish. Polystyrene, hazelnut shell, and toluene were mixed in the ratio of 2 wt.%: 8–48 wt.%: 50 to 90 wt.%. The composition was incubated for three days. Every 24 hours the composition was treated with ultrasound using ultrasound bath TECHMANN LABORANT L-22 Basic (ODA-Service LLC, Moscow, Russia) with 40 kHz frequency. Ultrasound is used to accelerate dissolution of substances. At the same time, a decrease in the proportion of sediment is observed. The dissolution of hard-to-dissolve substances goes faster. Processing by ultrasound leads to the formation of polystyrene coating on hazelnut shell particles during the modification process. 

The obtained solution was kept in a drying oven for 100–120 min at 80–95 °C. After that, the resulting material was ground in a planetary mill for at least 10 min and then sifted through a 64 µm sieve.

### 2.3. Obtaining a Composite 

Composites on the basis of polystyrene with the concentrations of modified filler of 10 %, 20%, 30%, 40%, and 50% by mass were made for the research. Polystyrene granules were previously ground for 3 min. Then, the modified filler and ground polystyrene were mixed in a planetary mill for 10 min. The resulting homogenized mixture was loaded into a mold with further heating to 165 °C for 60 min. After that, the samples were pressed under pressure of 110 MPa with load endurance for 5 min. The method of hot pressing of the samples allows for shear deformations, which leads to a uniform distribution of the filler in the melt. 

### 2.4. Research Methods

The grain size distribution was determined by laser diffraction using the Analysette 22 NanoTec plus equipment (Frisch, Idar-Oberstein, Germany). Hazelnut shell particles less than 64 μm in size were used in the study.

The density was determined by hydrostatic weighing based on Archimedes’ law: first the mass of the sample was determined in the air, then it was determined in a liquid with a known density (e.g., distilled water).

After weighing the samples in air and in liquid we found their density according to the Formula (1):

(1) $$p=\frac{m_{1}}{m_{1}-m_{2}}\left(p_{w}-\sigma \right)+\sigma$$

where *m*_1_—weight in the air; *m*_2_—weight in the water; $p_{w}$ = 0.998 g/cm^3^—water density at 20 °C; *σ* = 0.0012 g/cm^3^ air density.

The morphological properties of hazelnut shell powder and polystyrene composites were studied by scanning electron microscopy using the TESCAN MIRA 3 LMU equipment (Tescan, Brno, Czech Republic). Chromium was used as a conductive coating. To do this, a metal coating of chromium was applied to the samples under study by vacuum magnetron sputtering.

Using the NU-2 optical microscope (Karl Zeiss Jena, Germany), we viewed the magnified image of composites and the structure on the surface.

The flexural tests were carried out using the REM-100-A-1–1 testing machine (METROTEST Ltd., Bashkortostan, Russia). The length between the supports is 15 mm with a maximum load of 597 N. The flexural strength was determined by standard methods according to GOST R 57749-2017 (ISO 17138:2014). Studies were carried out for three-point flexural tests. Three-point flexural strength (MPa) was calculated by the Formula (2):

σ_f,m_ = 3*F*_m_·L/(2bh^2^) (2)

where *F*_m_—maximum load, N; L—distance between the bottom supports, mm; b—sample width, mm; h—average thickness of the sample, mm.

The test speed was 0.5 mm/min. The tests were carried out on five samples for each composition of the polystyrene composite, and then the average value was calculated.

The Vickers hardness of the surface was measured using the NEXUS 4504 hardness tester (INNOVATEST Europe BV, Maastricht, The Netherlands). The indenter was a four-sided Vickers diamond pyramid with a square base and an apex of 136° between the opposite faces. In all measurements the load was the same (200 g), and it was applied for a fixed time (15 s).

Water absorption was measured after soaking in distilled water for 1, 7, 20, and 30 days. Electronic scales were used. The following equation was used to calculate water absorption (% by weight):

(3) c=·100

where *m*_1_, *m*_2_—weight of the sample before and after soaking in water, respectively; c–percentage increase in weight.

Measurement of the wetting angle of the samples was carried out using Krüss DSA30 equipment (Krüss GmbH, Hamburg, Germany).

The VERTEX 70 FT-IR spectrometer (Bruker Optik GmbH, Germany) was used to study the functional groups of materials. Studies were carried out in the range 450–4500 cm^−1^. For the study, the method of diffuse reflection of infrared Fourier transform (DRIFT) was used. Hazelnut shell powder was pressed into a KBr tablet. The spectral resolution was 4 cm^−1^, and the number of scans was 32.

The thermal properties of the materials were determined by differential scanning calorimetry (DSC) using STA 449F1 Jupiter^®^ equipment (NETZSCH-Gerätebau GmbH, Selb, Germany). The studies were carried out in the temperature range from 20 to 1000 °C in an argon (Ar) atmosphere. The heating rate was 10 °C/min. The mass of the test sample was 10 mg. The studies were carried out in an atmosphere of argon gas (Ar).

The crystal structure was studied by X-ray diffraction using an ARL X’TRA (Thermo Fisher Scientific SARL, Ecublens, Switzerland). X-ray patterns were taken in the range of 2θ–4–64° double reflection angles on filtered CuKa radiation with Ni-filter, with tube voltage—20 kV, tube anode current—8 mA, measurement limit from 1000 to 4000 imp/s, counter speed—4 deg/min, and angle marking—1°.

## 3. Results

### 3.1. Study of Hazelnut Shell Powder

**Figure 1:**
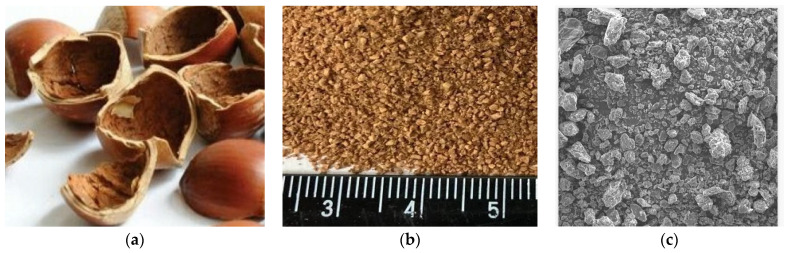
(**a**) Hazelnut shell; (**b**) hazelnut shell powder; (**c**) SEM image of hazelnut shell powder.

Figure 1 shows photos of hazelnut shells used before and after grinding. The cross-sectional size of the hazelnut shell is 11–13 mm (Figure 1a). After grinding the shells, fine powder is formed (Figure 1b). At high magnification using scanning electron microscopy, it can be noted that after grinding, the particle size of the powder ranges from 1 to 15 μm (Figure 1c). The particles have irregular, elongated shapes. It is noticeable that some particles consist of several layers and have a scaly shape.

**Figure 2:**
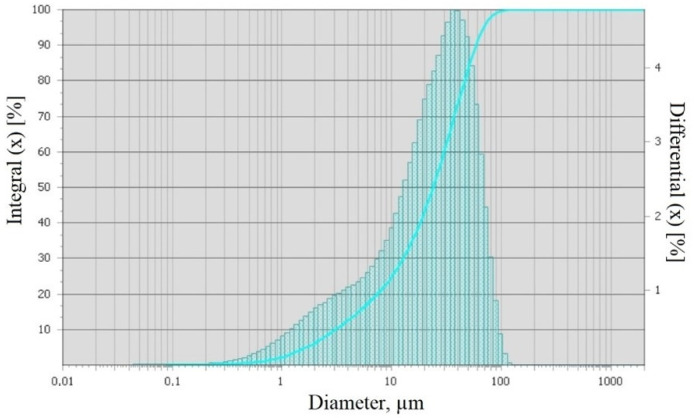
Fractional composition of hazelnut shell powder.

Grain size analysis of walnut shell powder was carried out using the method of laser scattering on a diffraction microanalyzer. Figure 2 shows the fractional composition of the obtained hazelnut shell powder.

The analysis of hazelnut shell powder granulometry data showed that the particles ranged from 0.01 to 120 μm, with most of the particles ranging in size from 1 to 45 μm (Figure 2). The modal particle diameter was 37.79 μm and the specific surface area was 8654 cm²/cm³. The granulometry data indicate strong aggregation of hazelnut shell powder particles, since before the study the powder was sifted through a sieve with a mesh size of 64 µm in diameter.

**Figure 3:**
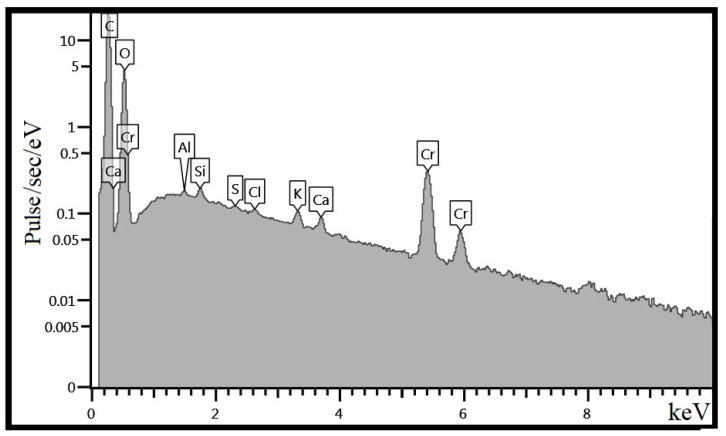
Spectrum of energy dispersive analysis of hazelnut shell powder.

The elemental composition of hazelnut shell powder was determined by energy dispersive microanalysis. The results are shown in Table 3 and in Figure 3. As a result of the morphological analysis, it was found that the filler consists mainly of carbon (C) and oxygen (O) atoms. All other elements are present in small amounts, which can be considered as impurities.

**Figure 4:**
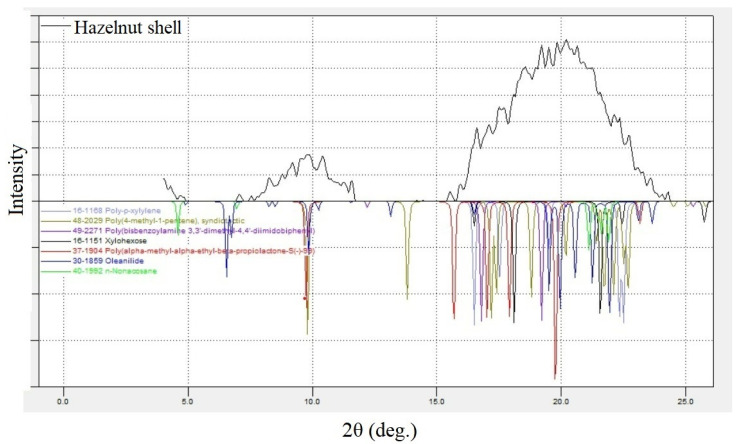
Powder X-ray diffraction pattern of hazelnut shells (the top of the drawing figure). The lower part of the figure is a comparison of X-ray diffraction patterns from the PDF file cabinet.

X-ray phase analysis was performed to determine the amorphous-crystalline structure of hazelnut shell powder. It is aimed mainly at determining the qualitative and semi-quantitative characteristics of raw materials. Crystals of each individual compound give a specific X-ray diagram with characteristic values of interplanar distances and a certain intensity of the corresponding reflections. Qualitative phase analysis of the composition is carried out by comparing the interplanar distances and their intensities obtained by decoding the given X-ray diffraction diagram. Figure 4 shows a powder X-ray diffraction pattern of hazelnut shells.

The analysis of the X-ray pattern (Figure 4) showed that the filler has a pronounced amorphous character. Two amorphous halos were recorded on the X-ray pattern at angles in the region of 7–12° and 15–24°. The X-ray diffraction patterns were interpreted using the Powder Diffraction File (PDF) database (file cabinet) of powder diffraction data. X-ray phase studies revealed the content of such substances as poly-p-xylylene (PDF card №16–1168), poly(4-methyl-1-pentene) (PDF card №48–2029), and poly(bisbenzoylamine 3,3′-dimethyl-4,4′-diimidobiphenyl) in the largest amounts, as well as others, in the sample (Figure 4).

### 3.2. Study of Modified Hazelnut Shell Powder

In order to create composite materials based on polymers, it is necessary to solve the problems of uniform distribution of the filler in the volume of the polymer matrix. For this purpose, the method of modifying hazelnut shell powder particles by polystyrene was developed to create a polystyrene coating on the surface of the hazelnut shell. In order to evaluate the creation of polystyrene coating on the surface of hazelnut shells, the wetting ability of the surface of the original and modified powder was studied. The wetting ability of the modified and unmodified filler was evaluated by the sitting drop method; wetting was performed with distilled water and diiodomethane. Initial values were extrapolated at zero time.

**Figure 5:**
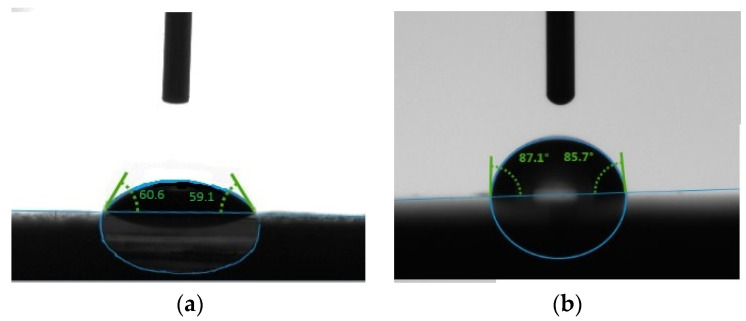
Photos of the water drop on the unmodified (**a**) and modified fillers (**b**).

The results of wetting angle measurements are presented in Table 4. Figure 5 shows photos of the water drop on the pressed samples of the original and modified filler.

It can be noted that the modification of the filler affected the increase in the contact angle of wetting with water from θ=60.16±1.03° to θ=87.02±1.10°. It can also be noted that the modification of the filler affected the increase in the contact angle of wetting with water from *θ* = 60.16 ± 1.03° to *θ* = 87.02 ± 1.10°. Thus, the modified filler became less hydrophilic compared to the unmodified one; namely, the hydrophilicity decreased by 44.64%. It should be noted that after modification, the indicators of the wetting angle of the filler are close to the indicators of the wetting angle of pure polystyrene, which suggests that a polystyrene coating was formed around the particles of the filler.

On the basis of data on the values of contact angles of wetting with water and diiodomethane, the values of free energy of the hazelnut shell powder surface were calculated before and after modification. Data on the free energy of the surface, dispersed, and polar parts are presented in Table 5.

The data on the surface free energy (Table 5) show an increase in the surface free energy, disperse, and polar readings of the modified filler as compared to the original powder. Thus, the surface energy increased by 30.3% compared to the unmodified filler. At the same time, the disperse component of surface tension is significantly greater than the polar part in both cases. It should also be noted that the surface free energy value of the modified filler is close to the literature data of pure polystyrene [38], which also suggests the formation of a polystyrene coating around the hazelnut particles.

**Figure 6:**
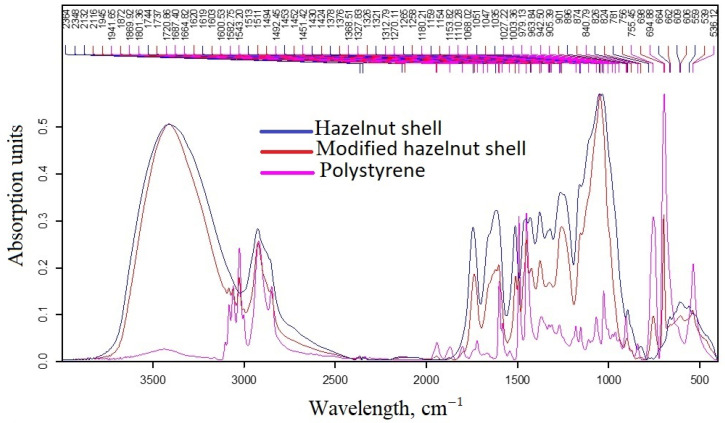
FTIR spectra of unmodified and modified fillers, as well as the FTIR spectrum of polystyrene.

FTIR spectroscopy was performed to confirm the creation of a polystyrene shell on the surface of hazelnut shell powder particles. Figure 6 shows FTIR spectra of modified and unmodified fillers of hazelnut shell powder, as well as the spectrum of pure polystyrene. The series of bands from ν = 2364–2116 cm^−1^ refer to the characteristic absorption bands of hazelnut [39]. Intense and broad bands in the region of 755 and 694 cm^−1^ are characteristic of polystyrene [40]. Several bands of medium and low intensity in the region of 1664 and 840 cm^−1^ are also characteristic frequencies for this polymer. Similarly, polystyrene absorption bands are expressed by peaks in the region of ~2848 cm^−1^, ~2924 cm^−1^, and ~3001 cm^−1^ [40].

Since on the FTIR spectrum of modified hazelnut shell powder we can observe bands characteristic of polystyrene, which are absent on the FTIR spectrum of unmodified hazelnut shell powder, namely peaks in the regions of 756 cm^−1^, 2923 cm^−1^, and 3002 cm^−1^, then we can conclude that a polystyrene coating has formed on the hazelnut particles.

### 3.3. Mechanical Characteristics of Composites

**Figure 7:**
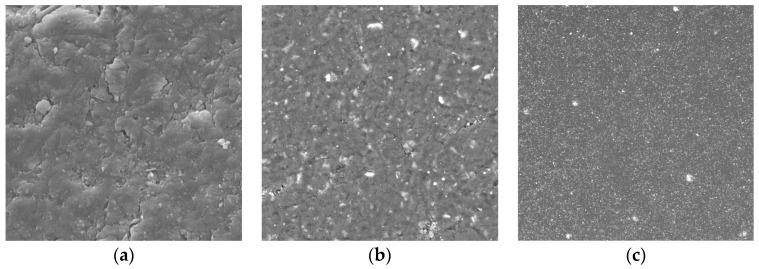
SEM images of the surface of the composite sample containing 30 wt.% of the modified filler. (**a**) 2 µm; (**b**) 20 µm; (**c**) 200 µm.

In order to study the distribution of the modified filler in polystyrene composite, the surface structure was examined using high magnification. Petrographic analysis or the method of visual examination is carried out in order to study the sample surface microstructure and to analyze the content of different phases. The surface analysis was carried out by means of the polished section preparation. The obtained images are shown in Figure 7.

The analysis of the obtained SEM images of the surface of the composite sample containing 30 wt.% the modified filler (Figure 7) showed that the sample was characterized by uniform distribution of particles of modified hazelnut shells in the entire volume of the polystyrene matrix, which indicates good adhesion at the interface between the particle-polymer phases. No cracks, layer separations, or chips were detected.

The physical and mechanical properties of composites based on polystyrene and modified hazelnut shell powder were studied (Table 6). The concentration of the filler was from 10 to 50 wt.%. The addition of the modified powder to the composite contributes to an increase in the density of the material (Table 6). This is due to the fact that the density of the modified powder is slightly higher compared to the density of polystyrene. In spite of this, the density of composites is rather low.

**Figure 8:**
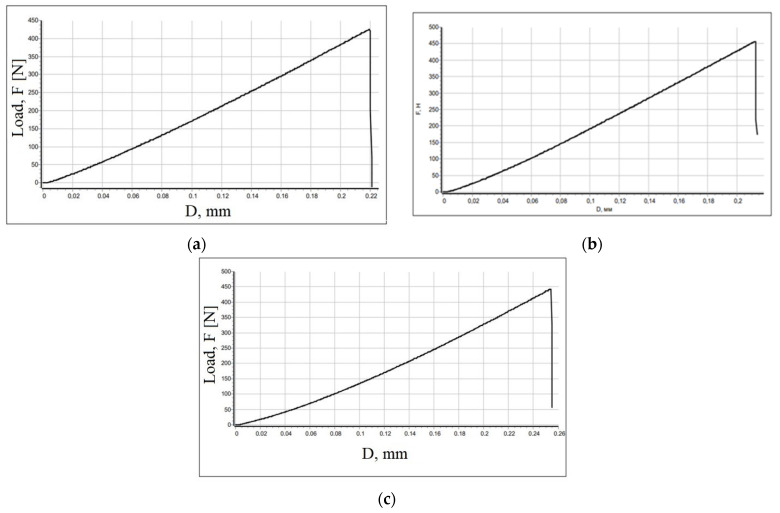
Load-time curves for composites containing a modified filler, wt.%: (**a**) 20; (**b**) 30; (**c**) 40.

The ability of the composite to resist internal stresses under external load was studied. The introduction of the modified filler in a certain ratio contributed to an increased strength of the materials. The tests were conducted at a maximum load of 497 N. Figure 8 shows the strength test graphs for different polystyrene to filler ratios.

The maximum deformations during flexural tests are: 0.22 mm for the composite containing the modified filler in the amount of 20 wt.%; 0.21 mm for the composite containing the modified filler in the amount of 30 wt.%; 0.25 mm. for the composite containing the modified filler in the amount of 40 wt.%; 0.22 mm for the composite containing the modified filler in the amount of 20 wt.%; 0.21 mm for the composite containing the modified filler in the amount of 30 wt.%; and 0.25 mm for the composite containing the modified filler in the amount of 40 wt.%. Consequently, the strongest composition has the composite with the ratio of polystyrene 70 wt.% / modified hazelnut shell 30 wt.%. Vickers hardness tests determine the ability of the material to resist plastic deformation caused by a specific standard source. Pure polystyrene shows the values that are 24% lower than polystyrene with the addition of filler in various ratios (Table 6). Consequently, the introduction of the filler improves the strength characteristics of the composites.

**Figure 9:**
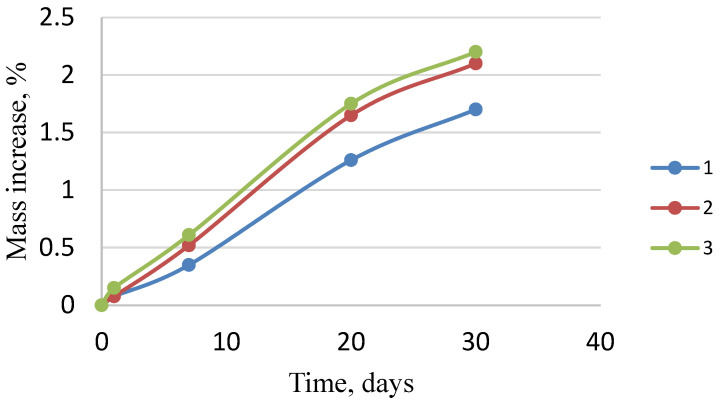
The curve of weight increase dependence on the time the composites containing modified filler (wt.%: (1) 20; (2) 30; (3) 40) are in water.

Figure 9 shows the graph of the dependence of mass gain on the time the samples were in water. With a significant increase in the filler introduced (more than 50 wt.%), a sharp decrease in the water absorption indicator is observed. This can be caused by the porous structure of the hazelnut shell particles or an increase in the interfacial void in the matrix. This is what leads to a decrease in the physical and mechanical characteristics (Table 6).

As a result of experiments, it was established that composites had optimal physical, mechanical, and operational properties at the following ratio: polystyrene 60–80 wt.%, modified hazelnut shell powder 20–40 wt.%. With the introduction of more than 90 wt.% of polystyrene, the flexural strength and Vickers hardness at 200 g were quite low, and thus, the strength of such materials is not satisfactory (Table 6). These samples are also characterized by a small percentage of hazelnut shells; therefore, the resulting material is of pale, unsaturated color. With polystyrene content less than 50 wt.%, the water absorption rate increases, and the physical and mechanical parameters —flexural strength and Vickers hardness at a load of 200 g—also decrease significantly (Table 6).

### 3.4. Thermal Properties of Composites

**Figure 10:**
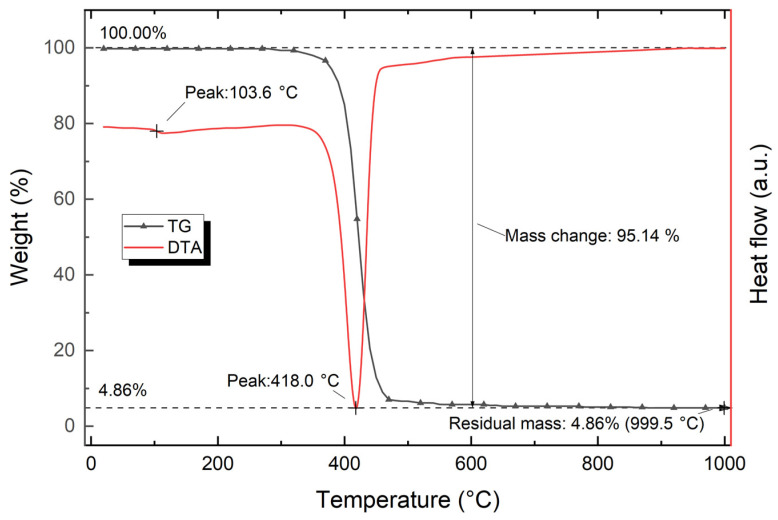
TG and DTA curves of polystyrene.

Figure 10 shows the data of thermal analysis of polystyrene used (TG and DTA curves of polystyrene).

The analysis of the TG curve showed that after reaching 999.5 °C, the total weight loss is about 95.14%. The TG curve has the appearance of a typical single-step decomposition curve. In the temperature range of 295.0–624.0 °C, there is a loss of about 95% of the initial weight. This interval contains the endothermic peak at 418.0 °C with an influence interval in the range of 329.0–455.0 °C. The weight loss is predominantly due to the decomposition of polystyrene, which is indicated by the one-step TG curve and the endothermic nature of the peak at 418.0 °C. The peak at 103.6 °C corresponds to the glass transition temperature of polystyrene.

**Figure 11:**
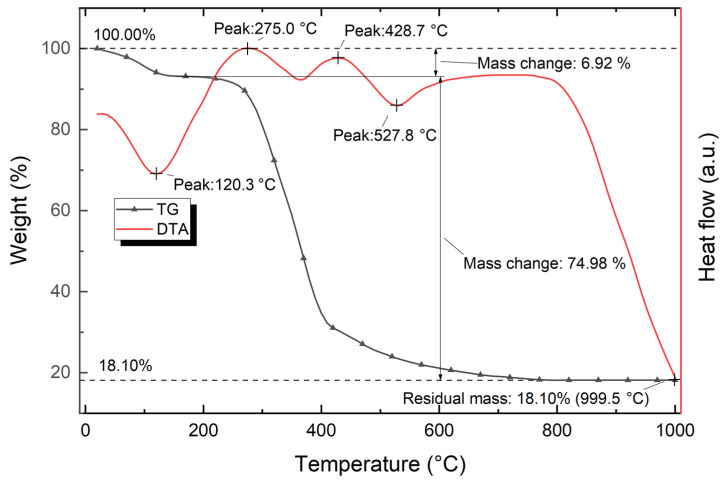
TG and DTA curves of hazelnut shell powder.

Figure 11 shows the data of thermal analysis of modified hazelnut shell powder (TG and DTA curves of modified hazelnut powder).

The analysis of the TG curve of the modified hazelnut powder (Figure 11) showed that after reaching 999.5 °C, the total weight loss is about 81.90%. The TG curve has a typical two-step decomposition curve with formation of relatively stable substances, which is evidenced by a plateau in the temperature range of 150.0–200.0 °C, where there is practically no change in the weight of the composite. There is a loss of about 6% of the initial weight in the temperature range of 25.0–150.0 °C, which corresponds to the first step in the two-step curve. Due to the fact that the peak of 120.3 °C falls in this range, with a pronounced endothermic effect, the weight loss is due to the adsorbed water being removed.

In the temperature range of 200.0–744.8 °C, three peaks of different natures are observed, the combined effect of which leads to a loss of ≈75% of weight. The first exothermic peak is at 275.0 °C; its influence interval is in the range of 200.0–364.0° C. This peak corresponds to the beginning of thermal decomposition of the hazelnut powder, which corresponds to the beginning of intense weight loss. The third exothermic peak is at 428.7 °C; its influence interval is between 364.0 and 483.4 °C. This peak also corresponds to the thermal decomposition of the hazelnut powder, but the weight loss at this interval is less intense compared to the previous one.

The last endothermic peak is at 527.8 °C, with an influence interval in the range from 483.4 °C to 744.8 °C. This peak corresponds to the end of the thermal decomposition of the hazelnut powder; at this interval the weight loss is much less intense compared to the primary weight loss. The end point of this interval corresponds to a maximum weight loss of 81.90%; further changes are minimal.

The thermal analysis of polymer composites was carried out to estimate the upper limits of working temperatures and to study physical transitions occurring in the polymer composite under the influence of temperatures accompanied by endo- and exo-effects. The results of the above-mentioned residual weight analysis at different temperatures for polymers with different hazelnut powder content are presented in Table 7. Pure polystyrene remains relatively stable up to a temperature of 300 °C, after which a rapid weight loss occurs between 400 °C and 500 °C due to the breakdown of the polymer chains and consequent decomposition of the polymer.

**Figure 12:**
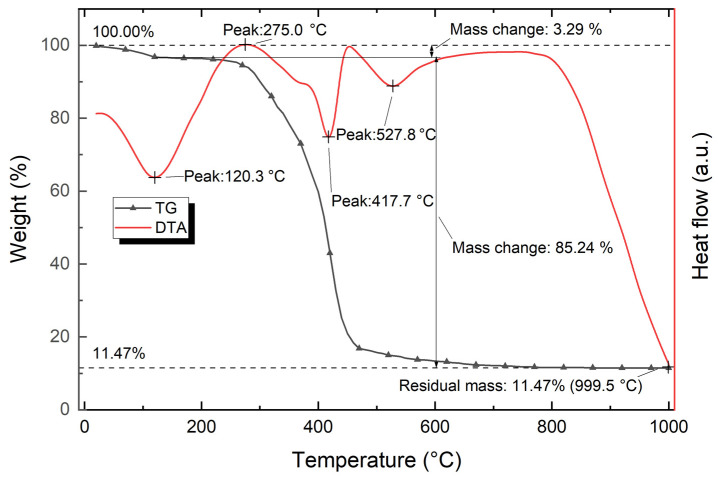
TGA and DTA curves for the composite containing 50 wt.% of modified hazelnut shell powder.

Figure 12 shows the data of thermal analysis of the polymer composite based on polystyrene with the addition of modified hazelnut shell powder (TG and DTA curves of the composite containing 50 wt.% of modified hazelnut shell powder).

The analysis of the TG curve of the composite showed that after reaching 999.5 °C, the total weight loss is about 88.53%. The TG curve, as in the case of the TG curve for hazelnut powder, has a typical two-step decomposition curve with the formation of relatively stable substances, which is evidenced by a plateau in the temperature range between 120.0 °C and 200.0 °C, where there is almost no change in the weight of the composite. In the temperature range of 25.0–150.0 °C, there is a loss of about 3.29% of the initial weight, which corresponds to the first step in the two-step curve. Due to the fact that in this interval there is a peak of 120.3 °C, with a pronounced endothermic effect, the weight loss is similar to the case of hazelnut powder, where the adsorbed water is removed.

In the temperature range between 200.0 °C and 744.8 °C, three peaks of different nature are observed, the combined effect of which leads to a loss of ≈85% of the weight. The first exothermic peak occurs at 275.0 °C; its influence interval lies in the range 200.0–364.0 °C. This peak is consistent with the onset of thermal composite, which corresponds to the beginning of intense weight loss. The second peak is endothermic in nature and occurs at 417.7 °C, with its influence interval ranging from 377.9 °C to 452.8 °C. This peak also corresponds to the thermal decomposition of the composite; the weight loss is of maximum intensity and predominantly accounts for the weight loss of polystyrene (having a similar peak with maximum weight loss).

The last endothermic peak occurs at 527.8 °C with an influence interval in the range between 452.8 °C and 744.8 °C. This peak corresponds to the end of thermal decomposition of the composite; at this interval the weight loss is much less intense compared to the primary weight loss. The end point of this interval corresponds to the maximum weight loss of 88.53%; further changes are minimal.

Since the maximum weight loss allowed for polymer compositions is 5%, the upper limit of the operating temperature range for the polymer is 265.0–376.0 °C, depending on the percentage of hazelnut shell powder in the composite.

The developed technology for the synthesis of composites based on polystyrene and softened hazelnut shell powder makes it possible to use this composite material to create outdoor and garden furniture. Compared with the known polymer composites based on vegetable raw materials, the proposed composite has a number of advantages. For example, a hybrid composite based on polypropylene with basalt/hazelnut shell fillers is known [33]. For its synthesis, 5 wt.%, 7.5 wt.%, and 10 wt.% by weight of basalt fibers and crushed hazelnut shells were added to the polypropylene matrix. Compared to the composite we offer, it has low physical and mechanical characteristics, a high water absorption rate, and a low aesthetic level for perception. There is known work on the creation of a polymer composite for chipboard from waste wood and polystyrene foam [41]. However, the addition of wood waste leads to a significant increase in the water absorption of the composite and to the impossibility of using it to create street furniture compared to the composite we offer.

## 4. Conclusions

The technology of obtaining composite material based on polystyrene and hazelnut shell powder has been developed. The creation of polystyrene cover on the surface of the hazelnut shell powder has increased the corner angle of wetting with water from θ=60.16±1.03° to θ=87.02±1.10°, which has considerably raised the degree of hydrophobicity of the filler. The use of preliminary modification of hazelnut shell powder particles allowed the introduction of up to 50 wt.% of the filler. The developed compositions and the method of receiving a polymeric composite allow to receive a material with high physical and mechanical characteristics, a low parameter of water absorption, a low cost, and a high aesthetic level for perception, due to the rich color of the hazelnut shell being an imitation of dark wood. The upper limit of the operating temperature range for the composite is 265.0–376.0 °C, depending on the percentage of the hazelnut shell powder filling.

The optimal composition of the composite includes 60–80 wt.% of polystyrene and 20–40 wt.% of modified hazelnut shell powder. When the content of the modified filler is 30 wt.%, the composite has the following characteristics: density—1154 kg/m^3^, Vickers hardness at the load of 200 g—20.19, water absorption—2.1%, and flexural strength—20.41 MPa. The given composite material can find applications in the creation of outdoor and garden furniture because it has a beautiful, saturated color; additionally, it will help to solve the ecological problem of hazelnut shells utilization. Further research should be aimed at finding new plant wastes that can be used to create polymer composites.

## Figures and Tables

**Table 1:** Properties of Polystyrene 525.

Grade	Density g/cm^3^	Melt Flow Index at 200 °C at 5 kg load g/10 min.	Melting Point °C
525	1.12	9.0 ± 2.0	160–170

**Table 2:** Properties of Toluene.

Formula	Density g/cm^3^	Molar Mass g/mol	Boiling Point °C
C_7_H_8_	0.87	92.14	110.6

**Table 3:** Elemental composition of hazelnut shell powder filler.

Atom	C	O	Al	Si	S	Cl	K	Ca
Weight %	69.01	30.46	0.04	0.09	0.03	0.04	0.16	0.16

**Table 4:** Data on the wetting angle of hazelnut shell powder.

Sample	Contact Angles of Wetting with Water, ° (Degrees)	Contact Angles of Wetting with Diiodomethane, ° (Degrees)
Original powder	60.16±1.54	30.36 ± 1.05
Modified powder	87.02±1.47	22.45±1.17

**Table 5:** Data on surface free energy, dispersed, and polar parts of hazelnut shell powder.

Sample	Disperse Part [mN/m]	Polar Part [mN/m]	Surface Free Energy [mN/m]
Original powder	32.82±1.07	2.08 ± 0.79	34.90 ± 1.36
Modified powder	40.93±2.96	4.57±1.01	45.50 ± 2.04

**Table 6:** Physical and mechanical characteristics of polymer composite.

Indicator	Content of Modified Filler, wt.%				
	10	20	30	40	50
Density ρ, kg/m^3^	1070	1074	1154	1173	1208
Vickers hardness at the load of 200 g	16.16	18.45	20.19	19.95	17.6
Water absorption, %	1.4	1.7	2.1	2.2	2.8
Flexural strength, MPa	13.96	16.67	20.41	20.7	14.17

**Table 7:** Residual weight in composites with modified hazelnut shell powder.

Temperature, °C	Content of Modified Hazelnut Shell, wt.%					
	0	10	20	30	40	50
100	99.77	99.37	98.97	98.57	98.17	97.76
200	99.77	99.10	98.42	97.75	97.07	96.40
300	99.31	97.49	95.66	93.84	92.00	90.17
400	84.97	79.95	74.91	69.87	64.81	59.75
500	6.62	8.43	10.24	12.05	13.87	15.69
600	5.74	7.26	8.79	10.32	11.86	13.39
700	5.30	6.66	8.03	9.40	10.77	12.15
800	5.08	6.38	7.68	8.98	10.28	11.59
900	4.86	6.18	7.50	8.82	10.15	11.48
1000	4.86	6.18	7.50	8.82	10.15	11.48

## Data Availability

Data sharing not applicable.
